# Bildgebung des Beckenbodens: Die Sicht der Gynäkologie

**DOI:** 10.1007/s00117-023-01215-7

**Published:** 2023-10-03

**Authors:** Teresa Anzböck, Dominique Koensgen

**Affiliations:** Klinik für Gynäkologie und gynäkologische Onkologie, Sektion für Urogynäkologie, Universitätsklinikum Bonn, Venusberg-Campus 1, 53127 Bonn, Deutschland

**Keywords:** Beckenbodendysfunktion, Diagnostik, Sonographie, Descensus genitalis, Inkontinenz, Pelvic floor dysfunction, Diagnostics, Ultrasound, Pelvic organ prolapse, Incontinence

## Abstract

**Hintergrund:**

Funktionsstörungen des Beckenbodens stellen ein gehäuft auftretendes Krankheitsbild der Frau dar.

**Ziel der Arbeit:**

Beschreibung des Stellenwerts der Sonographie in der urogynäkologischen Untersuchung und Bildgebung des Beckenbodens.

**Material und Methoden:**

Analyse und Zusammenfassung der aktuellen Empfehlungen und Literatur zur Durchführung der Pelvic-Floor-Sonographie.

**Ergebnisse:**

Die Pelvic-Floor-Sonographie ist eine meist zur Verfügung stehende, für die Patientinnen minimal belastende, dynamische und Real-time-Bildgebung des Beckenbodens, die eine funktionell-morphologische Beurteilung der Anatomie ermöglicht.

**Diskussion:**

Die Sonographie des Beckenbodens hat in der präoperativen Diagnostik sowie im postoperativen Komplikationsmanagement einen hohen Stellenwert.

## Abstract (en)

**Background:**

Pelvic floor dysfunction is common in women.

**Objectives:**

To describe the role of ultrasound in the urogynecological examination and imaging of the pelvic floor.

**Materials and methods:**

Analysis and summary of current recommendations and literature on the role of pelvic floor ultrasound.

**Results:**

Pelvic floor ultrasound is a dynamic and real-time imaging modality. It is readily available, allows for a realistic assessment of anatomy and morphology, and poses minimal patient burden.

**Conclusions:**

Pelvic floor ultrasound is of great value in preoperative diagnostics as well as in the postoperative management of complications.

Neben den etablierten deskriptiven Untersuchungsmethoden der urogynäkologischen Diagnostik wie der klinischen Deszensusbeurteilung nach dem Pelvic Organ Prolapse Quantification System (POP-Q-System) und der urodynamischen Messung hat die Sonographie in den letzten Jahren einen festen Stellenwert in der Diagnostik von Funktionsstörungen des Beckenbodens erhalten.

Die Sonographie des Beckenbodens hat ihren Ursprung in den 1980er-Jahren, als es zum vermehrten Einsatz von Ultraschall in der Gynäkologie kam. Initial wurde die Perinealsonographie zur Beurteilung von Blasen- und Urethrafunktion eingesetzt. Im Jahr 1990 wurde die Introitussonographie als neue Methode in der Blasenfunktionsdiagnostik vorgestellt, und 2006 erfolgte schließlich die Zusammenführung aller Methoden unter dem Konzept der Pelvic-Floor-Sonographie [8, 9].

## Pelvic-Floor-Sonographie

Die sonographische Untersuchung ermöglicht eine dynamische Darstellung und funktionell-morphologische Beurteilung der Anatomie des Beckenbodens mit seinen 3 Kompartimenten. Sie gewährleistet eine wirklichkeitsnahe und physiologische Visualisierung der Beckenbodenregion, angefangen von Blase und Urethra über Vagina, Uterus und Douglas-Raum bis hin zum Rektum und dem Analsphinkter, sowohl in Ruhe als auch unter Belastung (Valsalva; [12]). Zur sonographischen Beurteilung des Beckenbodens stehen neben der gewohnten endosonographischen Transvaginalsonographie auch die Introitussonographie und Perinealsonographie zur Verfügung. Alle Methoden gemeinsam werden unter dem Begriff *Pelvic-Floor-Sonographie* zusammengefasst [9].

**Figure 1:**
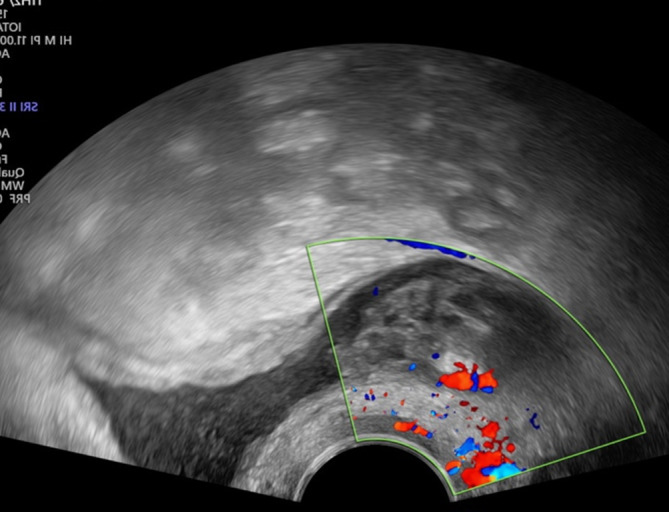

Die Transvaginalsonographie ermöglicht neben der klinischen Spekulumuntersuchung die Darstellung von nicht bzw. nicht nur durch den Beckenboden bedingten Ursachen urogynäkologischer Symptome. So kann beispielsweise eine Drangsymptomatik auch durch Urethra- oder Blasendivertikel, Blasentumoren, Raumforderungen des kleinen Beckens wie Uterusmyome oder Zysten in der Vaginalwand bedingt sein (Abb. 1; [12]).

Bei der Perinealsonographie erfolgt die externe sagittale Applikation einer Abdominalsonde im Bereich des Introitus vaginae mit einem möglichst geringen Auflagedruck, um einer Kompression des darunter gelegenen Gewebes vorzubeugen [12, 15]. Die Perinealsonographie ermöglicht insbesondere die Darstellung von Symphyse, Blase und Urethra, sodass die Blasenhalsregion mit ihren funktionellen Veränderungen beurteilt werden kann [6].

Die Introitussonographie stellt ein bildgebendes Verfahren dar, mit dem alle 3 Kompartimente des Beckenbodens erfasst werden und eine funktionell-morphologische Abklärung von Beckenbodenfunktionsstörungen wie Senkungszustände oder Inkontinenz erfolgen kann. Der Ultraschall ist das bildgebende Verfahren mit der höchsten Aussagekraft bezüglich der Darstellung einer Inkontinenz und Senkung sowie deren wechselseitiger Beeinflussung [15].

## Methodik

Die Pelvic-Floor-Sonographie sollte in Steinschnittlage am gynäkologischen Stuhl erfolgen. Der Schallkopf wird zwischen den gespreizten Labien auf das Perineum und den Introitus vaginae aufgesetzt. Die Perinealsonographie wird mit einer Curved-Array-Sonde mit einer Ultraschallfrequenz von 3,5–5 MHz durchgeführt, wohingegen bei der Introitussonographie ein Vaginalschallkopf mit einer Frequenz von 5–9 MHz verwendet wird. Die Ausrichtung des Schallkopfes sollte entsprechend der Körperachse erfolgen. Die Bilddarstellung wurde für Studien und Publikationen entsprechend der DEGUM-Empfehlung standardisiert: Kraniale Strukturen befinden sich oben und ventrale Strukturen rechts im Bild. Zu Beginn der Untersuchung wird eine Messung der Blasenfüllung empfohlen. Diese sollte idealerweise um die 300 ml betragen. Standardisierte Blasenvolumina ermöglichen einen Vergleich von prä- und posttherapeutischen Befunden [9, 10]. Der Beckenboden kann mit der Pelvic-Floor-Sonographie in 3 Ebenen dargestellt werden: sagittal, frontal und axial. Im Querschnitt kann der Beckenboden von der Symphyse ventral bis zum M. levator ani dorsal dargestellt werden. Zur Ultraschalldiagnostik aller Kompartimente sollte ein 2D-Ultraschall als Standard eingesetzt werden. Eine 3D-Sonographie kann als ergänzendes Verfahren in der morphologischen Beurteilung des M. levator ani und des M. sphincter ani sowie der Beckenorgane ergänzend eingesetzt werden. Als Referenzpunkt für die Messungen dient die Unterkante der Symphyse. Der knöcherne Bezugspunkt ermöglicht eine Beurteilung des Beckenbodens in Ruhe, beim Pressen und Husten sowie unter Beckenbodenkontraktion [10]. Dabei ist es entscheidend, dass der Schallkopf sowohl in Ruhe, als auch beim Valsalva-Manöver stets in der gleichen Position gehalten wird. Die *Cine-Loop-Funktion* am Ultraschallgerät ermöglicht es, die Ultraschallbilder der letzten Sekunden in Ruhe zu betrachten und die Dynamik der Beckenbodenstrukturen in den verschiedenen Phasen der Untersuchung besser zu analysieren [12]. Sie ermöglicht es ebenfalls, den Patientinnen die pathophysiologische Beziehung zwischen der Morphologie und der funktionellen Störung zu erklären und demonstrieren. Eine solche visuelle Darstellung kann auch als Biofeedback für die Patientinnen eingesetzt werden [10].

Während der verschiedenen Untersuchungsphasen sollte mit der Ultraschallsonde kein Druck auf das darunterliegende Gewebe ausgeübt werden, sodass die maximale Ausprägung des Deszensus erfasst werden kann [10]. So können dynamische Veränderungen wie Art und Schweregrad der Beschwerden funktionell und anatomisch erfasst, analysiert und dokumentiert sowie mit dem klinischen Untersuchungsbefund verglichen werden.

Die externe Applikation der Ultraschallsonde in Form der Introitussonographie hat gegenüber der Transvaginalsonographie den Vorteil, dass sondeninduzierten Einwirkungen auf die Anatomie des Beckenbodens während der Untersuchung weitestgehend vorgebeugt werden kann. So kann eine Verfälschung des Untersuchungsergebnisses durch die Sonde vermieden und der volle Umfang der Funktionsstörung des Beckenbodens bildgebend erfasst werden.

## Topographisch-funktionelle Beurteilung des Beckenbodens

Eine Descensus genitalis kann in den 3 Kompartimenten isoliert oder kombiniert auftreten. Bei Vorliegen eines Descensus genitalis sollte im Anschluss an die Transvaginal- und Introitussonographie auch stets eine sonographische Darstellung der Nieren zum Ausschluss einer Harnstauung durchgeführt werden [12].

Im vorderen Kompartiment können Messungen der Blasenwanddicke, der Restharnmenge sowie, in Bezug auf die Symphyse als stabile Struktur, die Länge der Urethra und die Lage des Meatus urethrae internus (MUI) erfolgen. Ebenfalls können qualitative Parameter wie der retrovesikale Winkel zwischen proximaler Urethra und Trigonum, eine Trichterbildung der proximalen Urethra (sog. Funneling) sowie die Position und Mobilität der Urethra und des Blasenbodens beurteilt werden [10, 12, 15]. Die Durchführung von Funktionstests wie Pressen, Husten und Kontraktion ist essenziell, um das Vorliegen einer Zystozele oder Urethrozele verifizieren zu können. Mithilfe der Inkontinenzprovokationstests können durch die Senkung ausgelöste Symptome sowie eine Belastungsinkontinenz während der Untersuchung repliziert werden.

Zystozelen können in 3 verschiedene Formen unterteilt werden: Pulsationszystozelen, Traktionszystozelen und kombinierte Traktions‑/Pulsationszystozelen. Die Unterscheidung erfolgt zwar primär klinisch, kann jedoch durch die Introitussonographie ergänzt werden. Bei der Pulsationszystozele handelt es sich um einen zentralen Defekt der endopelvinen Faszie. In der Spekulumeinstellung zeigen sich die Sulci vaginales laterales erhalten, die Rugae vaginales über der Zystozele jedoch verstrichen. Sonographisch zeigt sich der retrovesikale Winkel im Pressversuch spitz, das heißt im Vergleich zum Befund in Ruhe abnehmend. Durch die Senkung des Blasenbodens aufgrund des Fasziendefekts kann es zu Blasenentleerungsstörungen mit Restharnbildung, rezidivierenden Harnwegsinfekten und Drangsymptomatik kommen. Bei einer Traktionszystozele handelt es sich um einen lateralen Defekt mit Abriss des Arcus tendineus fascia pelvis von der Beckenwand. Dies bewirkt, dass sich in der Spekulumuntersuchung die Rugae vaginales über der Zystozele erhalten zeigen. Die Sulci vaginales laterales zeigen sich dahingegen bei einer Traktionszystozele verstrichen. Sonographisch zeigt sich dies in einem zunehmenden oder gleichbleibenden retrovesikalen Winkel unter Belastung. Bei Vorliegen sowohl eines lateralen als auch eines zentralen Defekts spricht man von einer kombinierten Traktions‑/Pulsationszystozele. Hierbei zeigt sich in der Sonographie meist ein unveränderter retrovesikaler Winkel unter Valsalva-Manöver [12].

**Figure 2:**
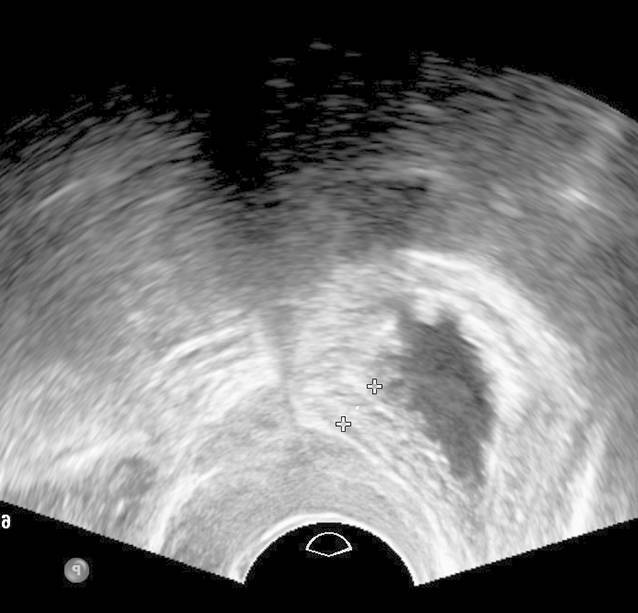

**Figure 3:**
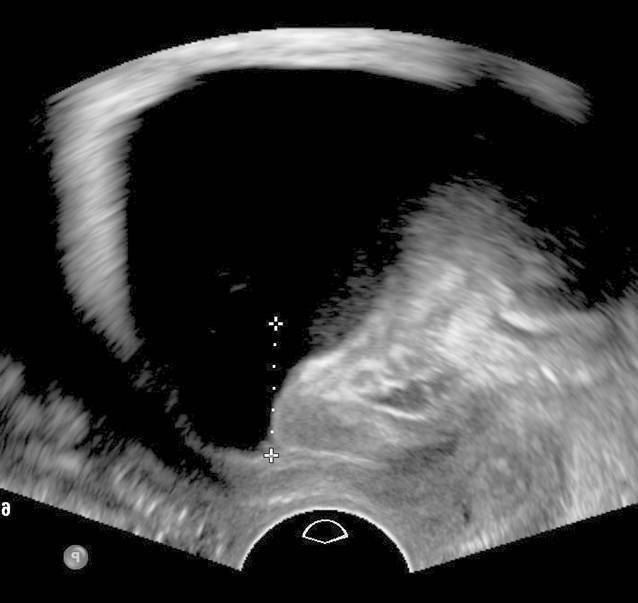

Die Introitussonographie ermöglicht es, die Urethralänge und die Mobilität der Harnröhre darzustellen. Im Fall einer Harndrangsymptomatik sollte die Blasenwand sonographisch beurteilt werden, um lokale Pathologien wie Blasenwanddefekte (Divertikel) als Ursache des Harndrangs auszuschließen. Eine pathologisch verdickte Blasenwand von ≥ 5 mm ist weiter abklärungsbedürftig. Eine asymmetrische Blasenwandverdickung kann ein Hinweis auf das Vorliegen eines Blasentumors sein. Eine symmetrische Verdickung der gesamten Blase kann hingegen mit einer überaktiven Blase/Detrusorüberaktivität oder Blasenauslassobstruktion (z. B. infolge einer Meatusstenose oder Harnröhrenstriktur) einhergehen (Abb. 2; [5, 11, 15]). Bei Patientinnen mit einer überaktiven Blase sollten zunächst morphologische Ursachen der Symptomatik, wie Senkungen im vorderen oder mittleren Kompartiment, Tumoren und Divertikel von Blase oder Urethra, ausgeschossen werden. Bei einer Senkung kann während des Pressens eine Lageveränderung der Blasenwand beobachtet werden, was für das Vorliegen einer Pulsationszystozele spricht. Eine dynamische Veränderung der Lage der Harnröhre unter Valsalva-Manöver ist hinweisgebend für eine Urethrozele. Ein Descensus urethrae kann in 2 verschiedenen Formen unterteilt werden: vertikal und rotatorisch. Beim vertikalen Deszensus kommt es zu einem Absinken des vesikourethralen Übergangs bei normalgestellter Urethra. Beim rotatorischen Descensus urethrae hingegen handelt es sich um eine kombinierte Senkung von Harnröhre und Blasenboden, sodass es zu einer Horizontallage der Urethra kommt (Abb. 3; [3]).

Die Beurteilung des periurethralen Gewebes in 2 Ebenen, ggf. auch mittels Farbdoppler, ist zum Nachweis bzw. Ausschluss von Pathologien, wie z. B. periurethralen Raumforderungen (Tumoren, Zysten, Abszesse, Varikosis oder Divertikel) geeignet.

Ein Deszensus des mittleren Kompartiments mit Tiefertreten des Uterus oder – nach Hysterektomie – des Vaginalstumpfes kann oftmals sonographisch genauer verifiziert werden als in der körperlichen Untersuchung. Bei der Spekulumeinstellung kommt es häufig zu einer Unterschätzung des Prolapsgrades, da ein nicht ausreichendes Nachgeben mit den Spekula während der Untersuchung die maximale Ausprägung der Senkung verhindert. Auch die Länge der Zervix sollte gemessen und im Verhältnis zum Corpus uteri beurteilt werden um eine Elongatio colli zu erfassen [15].

Im hinteren Kompartiment ermöglicht es die externe Ultraschallapplikation neben Rektozelen auch Enterozelen darzustellen. Der Inhalt der Enterozele kann sowohl aus Sigma als auch aus Dünndarm bestehen. Eine Unterscheidung ist mittels Sonographie nur bedingt möglich und benötigt eine weitere Schnittbildgebung zur genauen Einordnung [15].

Die 3D-Sonographie des hinteren Kompartiments ermöglich im Axialschnitt die direkte Beurteilung des M. levator ani, welcher häufig bei Geburten verletzt wird und eine wichtige Bedeutung in der Ätiologie des Descensus genitalis hat. Bei einer Avulsion des M. levator ani kommt es zu einer vollständigen Abtrennung des M. puborectalis vom Ramus inferior ossis pubis. Die Levatoravulsion kann sonographisch mit dem Nachweis einer Diskontinuität der Puborektalisschleife unter Beckenbodenkontraktion visualisiert werden [4, 15]. Ebenfalls kann im Axialschnitt die Beurteilung des M. sphincter ani erfolgen. Defekte der Mm. sphincter ani externus et internus zeigen sich nach höhergradigen Dammrissen als Verlust der Muskelkontinuität [15].

## Pelvic-Floor-Sonographie im präoperativen Management

Die Sonographie des Beckenbodens stellt eine einfach anwendbare und fast immer zur Verfügung stehende Untersuchungsmodalität dar. Sie kann die präoperative Diagnostik ergänzen und trägt zur Wahl der geeigneten Operationsmethode bei. Die nachfolgende Therapie kann durch einen präoperativ durchgeführten Ultraschall verbessert werden, indem dieser bereits vor dem Eingriff die Erkennung potenzieller Risikofaktoren für das postoperative Outcome ermöglicht [9, 15]. Zudem dient er als bildgebendes Dokumentationsverfahren zum Festhalten des präoperativen Befunds. Die Sonographie stellt eine ideale präoperative apparative Untersuchungsmodalität dar, da sie sowohl effizient und wirtschaftlich als auch schnell durchzuführen und für die Patientinnen wenig belastend ist [16]. Zudem sind sonographische Untersuchungen gut erlern- und standardisierbar, sie benötigen jedoch eine gewisse Expertise [1, 15].

**Figure 4:**
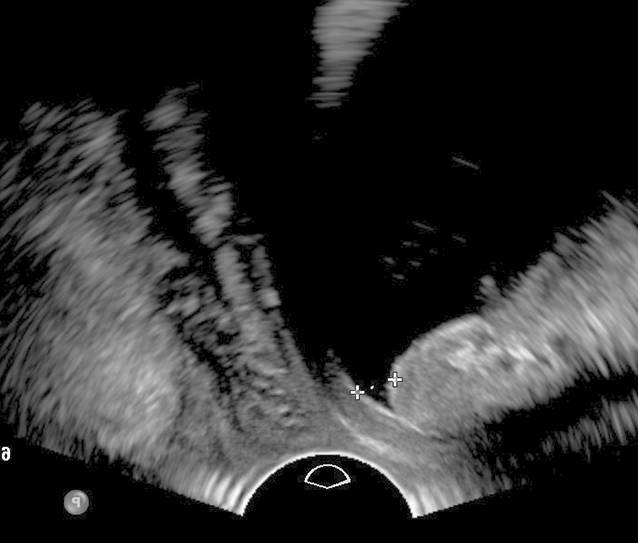

**Figure 5:**
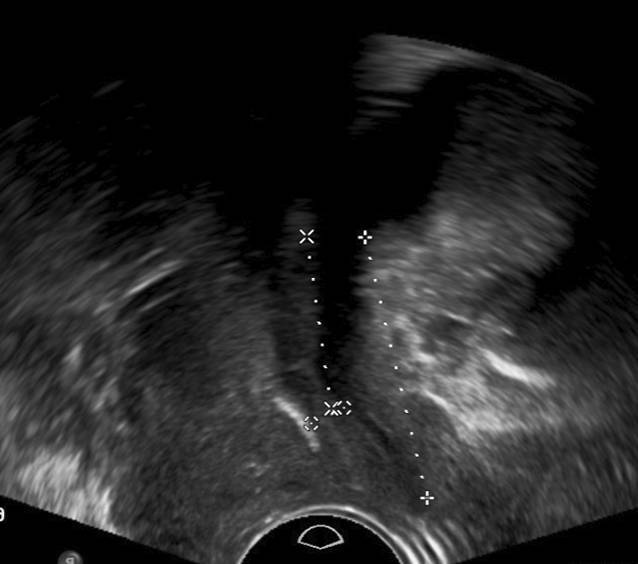

Präoperativ sollte die sonomorphologische Bestimmung der Urethralänge erfolgen, um die korrekte Platzierung eines Bands zu planen [7]. Die Position der Urethra sollte in 3 verschiedenen Zuständen geprüft werden (Ruhe, Valsalva und Beckenbodenkontraktion), um die Mobilität der Urethra beurteilen zu können. Dies ermöglicht die Identifikation einer hypermobilen oder starren Urethra, welche für die Erfolgschancen einer operativen Intervention von Relevanz sind. Das typische sonographische Korrelat einer Belastungsinkontinenz ist das Funneling (Abb. 4; [5]). Ein Funneling kann aber auch bei einer instabilen Urethra oder überaktiven Blase auftreten. Somit ermöglicht ein präoperativ durchgeführter Ultraschall klinisch okkulte Risikofaktoren (Urethradivertikel, hypermobile Urethra und Funneling) zu erkennen, welche mit dem Auftreten einer Rezidivharninkontinenz nach einer suburethralen Bandeinlage oder Kolposuspension assoziiert sind [10].

Vor einer geplanten chirurgischen Deszensusoperation sollte mithilfe des Ultraschalls eine Pathologie des Uterus und der Adnexen ausgeschlossen werden. Dies ist insbesondere bei einer geplanten uteruserhaltenden Operation von Bedeutung. Bei Vorliegen eines Descensus uteri kann eine präoperativ durchgeführte Sonographie eine Elongatio colli detektieren, welche bei einem organerhaltenden Vorgehen postoperativ zu einer Persistenz der Symptome führen kann [2]. Ebenfalls sollte präoperativ sonographisch das Risiko für das Bestehen einer lavierenden Inkontinenz evaluiert werden. Unter einer lavierenden Inkontinenz versteht man eine Inkontinenz, die durch einen Prolaps maskiert wird. Bei Belastung kommt es durch einen rotatorischen Descensus urethrae zu einem Abknicken der Urethra, sodass eine bereits vorhandene Inkontinenz kaschiert wird. Nach einer operativen Korrektur des Deszensus kann folglich die bereits präoperativ bestehende Inkontinenz symptomatisch werden [12].

## Pelvic-Floor-Sonographie im postoperativen Setting

**Figure 6:**
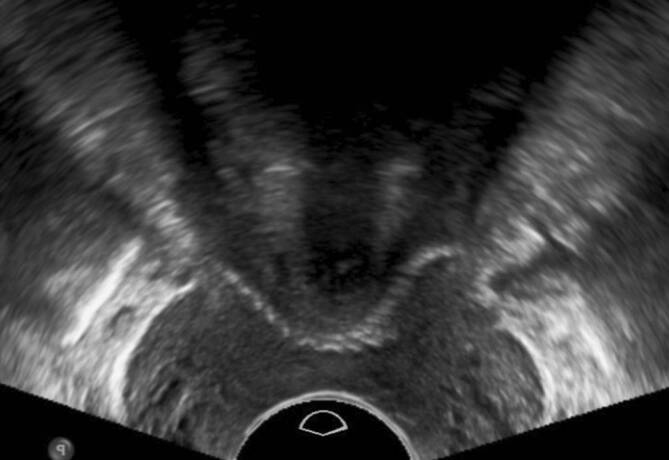

Die postoperative Sonographie des Beckenbodens ermöglicht es, die neuen anatomischen Verhältnisse und das Ergebnis der Operation zu überprüfen. In der Deszensuschirurgie werden in Deutschland häufig nichtresorbierbare synthetische Netzte eingesetzt, um bei unzureichendem Vorliegen von Eigengewebe dieses zu ersetzen oder zu unterstützen. Auch in der operativen Inkontinenzbehandlung wird häufig Fremdmaterial unter die Urethra gelegt, um eine Belastungsinkontinenz zu behandeln. Nach Einlage eines Implantats können Lage und Funktion des Fremdmaterials überprüft werden. Da sowohl in der Deszensuschirurgie als auch in der Inkontinenzchirurgie überwiegend Implantate aus Polypropylen verwendet werden, lassen sich diese echogenen Implantate besonders gut mittels Sonographie erfassen [14]. Das Verhalten des Implantats und dessen Auswirkung auf die umliegenden anatomischen Strukturen können sonographisch in Ruhe, unter Valsalva sowie bei Kontraktion des Beckenbodens beurteilt werden [10]. Nach jeder Einlage eines Tension-free-vaginal-tape(TVT)-Bands sollte eine Introitussonographie erfolgen, um die Lage des Bands sowie die Konfiguration zu beurteilen. In der Sagittalebene sollte das Band am Übergang des unteren zum mittleren Drittel der Harnröhre zur Darstellung kommen. In dieser optimalen Lage kann es seine Wirkung in der sog. „high pressure zone“ unter Belastung entfalten. Der Abstand des Bands zur Harnröhre sollte 3–5 mm betragen (Abb. 5). Eine fehlerhafte Lage kann entweder bei zu geringem Abstand die Blasenentleerung stören oder bei zu großem Abstand keine suffiziente Wirkung an der Harnröhre erzielen. Folglich kann es zu einer persistierenden Harninkontinenz kommen. Auch die Form des Bands kann im Ultraschall beurteilt werden. Diese lässt sich am besten in der Axialebene darstellen. Das Band sollte in einer C‑Form parallel zur Harnröhre liegen, ohne diese zu berühren oder einzuengen, da dies eine „de-novo overactive bladder“ (OAB), Dysurie oder Blasenentleerungsstörung begünstigen könnte. Auch die Symmetrie des Bands kann beurteilt werden, wobei eine symmetrische Lage erstrebenswert ist (Abb. 6; [9, 12]). Nach einer Kolposuspension kann das Operationsergebnis sonographisch anhand von Lage und Mobilität des Blasenhalses und des retrovesikalen Winkels beurteilt werden [10].

## Pelvic-Floor-Sonographie im postoperativen Komplikationsmanagement

Im Falle von postoperativen Komplikationen sollte eine sonographische Kontrolle zur Differenzialdiagnostik erfolgen. So können eine postoperative Blasenentleerungsstörung oder Drangbeschwerden durch eine Fehlposition des Implantats ausgelöst werden. In diesen Fällen kann eine operative Korrektur des Bands erfolgen, bevor es zu einem Einwachsen in das umliegende Gewebe kommt [7]. Bei korrekter Lage des Implantats kann ein Harnwegsinfekt, ein Hämatom oder eine postoperative Schwellung ursächlich für die Drangsymptomatik oder erschwerte Blasenentleerung sein [9]. Es sollten die Mobilität der Urethra sowie die Restharnmenge dokumentiert werden. Im Ultraschall lässt sich nach einer Schlingeneinlage zur Behandlung einer Belastungsinkontinenz bei einer zu straffen Bandlage ein Urethra-Kinking, also ein Abknicken der Urethra um die Schlinge, beobachten [12]. Bei einer postoperativen Blasenentleerungsstörung nach Kolposuspension sollte durch die Pelvic-Floor-Sonographie eine Überkorrektur ausgeschlossen werden. Bei einer Überkorrektur zeigt sich eine Elevationshöhe des MUI von ≥ 10 mm im Vergleich zur präoperativ gemessenen Ruheposition im Liegen. Der sonographische Befund kann damit das postoperative therapeutische Konzept wesentlich beeinflussen (Fadenentfernung vs. Observation vs. Zystozelenkorrektur; [10]). Bei Auftreten einer Rezidivharninkontinenz nach Kolposuspension lässt sich in der Ultraschalluntersuchung häufig ein Funneling oder einer persistierende Hypermobilität der Harnblase und Harnröhre darstellen [15]. Die Pelvic-Floor-Sonographie kann postoperativ auch im Rahmen der Fisteldiagnostik ergänzend zur Spekulumuntersuchung eingesetzt werden, da sich in manchen Fällen die Fistel sonographisch darstellen lässt [10]. Auch im Fall einer Rezidivsituation nach Prolapskorrektur sollte eine Pelvic-Floor-Sonographie erfolgen. So können beispielsweise Lage und Konfiguration eines eingesetzten Netzes oder Bands überprüft und die genaue Lokalisation des Rezidivs dokumentiert werden. Nach einer Mesh-Einlage im vorderen Kompartiment können 3 verschiedene Rezidivsituationen unterschieden werden: ventrales, apikales und globales Rezidiv. Beim ventralen Rezidiv kommt es zu einer Zystozelenbildung ventral des eingesetzten Netzes, während es beim apikalen Rezidiv zu einem Wiederauftreten der Zystozele oder des apikalen Deszensus kranial des Netzes kommt. Bei einem ventralen Rezidiv ist von einer Dislokation der kaudalen Netzarme auszugehen, wohingegen bei einem apikalen Rezidiv eine vermehrte Beweglichkeit des kranialen Netzanteils ursächlich ist. Bei einem globalen Rezidiv ist das gesamte Netz übermäßig mobil, sodass von einem kompletten Ausriss des Implantats an seinen Fixierungspunkten und einer damit einhergehenden völlig fehlenden wirksamen Verankerung auszugehen ist. Bei einem Rezidiv nach einer Netzeinlage im hinteren Kompartiment kann zwischen einem ventralen und dorsalen Rezidiv unterschieden werden. Auch eine Raffung des Fremdmaterials kann sonographisch dargestellt werden und für eine Fehlfunktion des implantierten Materials verantwortlich sein [13, 14].

## Fazit für die Praxis

- Die sonographische Beurteilung des Beckenbodens sollte in Ruhe, beim Pressen und Husten sowie unter Beckenbodenkontraktion erfolgen.
- Die Pelvic-Floor-Sonographie sollte vor jedem geplanten Eingriff am Beckenboden zur operativen Korrektur einer Inkontinenz oder eines Deszensus durchgeführt werden.
- Im Fall von postoperativen Komplikationen sollte eine Sonographie zur Differenzialdiagnostik durchgeführt werden.
- Bei Rezidivdeszensus nach vorangegangener Implantateinlage sollte eine sonographische Beurteilung der Anatomie und des Implantats erfolgen.
